# Supplementary material for: Impact of Hormone Replacement Therapy on Risk of Ovarian Cancer in Postmenopausal Women with De Novo Endometriosis or a History of Endometriosis
Source: Cancers (Basel). 2023 Mar 10;15(6):1708. doi: 10.3390/cancers15061708 (PMC10046182; doi:10.3390/cancers15061708)
Supplement: Supplementary file 1 [file cancers-15-01708-s001.zip › Supplemental Figure S5.pptx]

## Slide 1
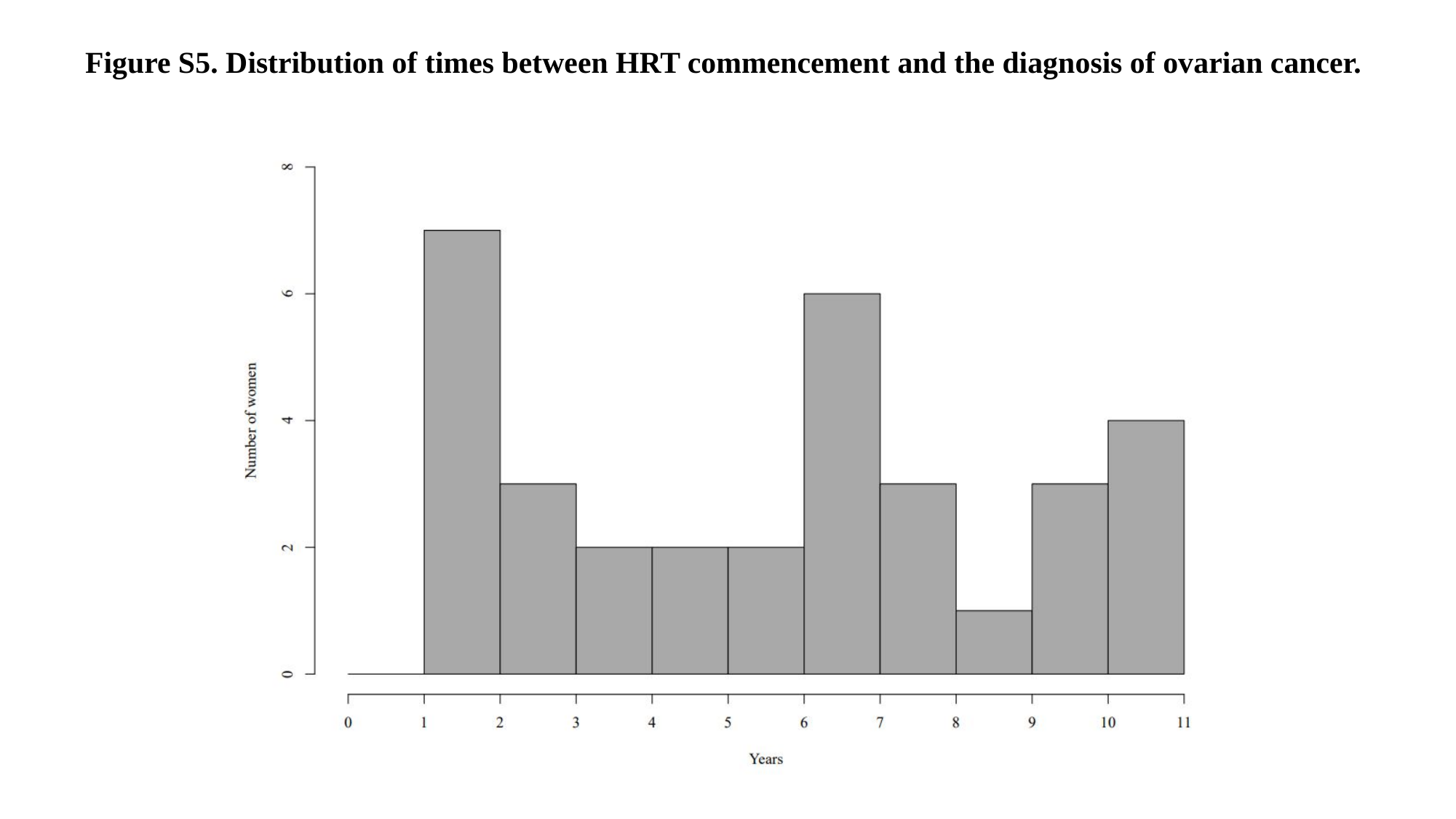

Figure S5. Distribution of times between HRT commencement and the diagnosis of ovarian cancer.
